# Elevated serum C-reactive protein level predicts a poor prognosis for recurrent gastric cancer

**DOI:** 10.18632/oncotarget.9910

**Published:** 2016-06-09

**Authors:** Fanming Kong, Fangfang Gao, Jun Chen, Rongxiu Zheng, Honggen Liu, Xiaojiang Li, Peiying Yang, Geli Liu, Yingjie Jia

**Affiliations:** ^1^ Department of Oncology, First Teaching Hospital of Tianjin University of Traditional Chinese Medicine, Tianjin, China; ^2^ Department of Pediatrics, Tianjin Medical University General Hospital, Tianjin, China

**Keywords:** recurrent gastric cancer, C-reactive protein, prognosis

## Abstract

**Backgrounds:**

High serum C-reactive protein (CRP) was found to be associated with poor prognosis in kinds of solid tumors, however, its role in the recurrent gastric cancer (RGC) is unknown. The present study aimed to explore the prognostic value of serum CRP in RGC patients.

**Methods:**

A total 72 RGC patients who underwent radical surgery from January 2005 to May 2008 were enrolled. The clinical, pathological and survival information were collected. The serum CRP level was measured when the recurrence was confirmed, and the association between serum CRP and clinicopathological characters was analyzed. The prognostic value of serum CRP for RGC was investigated.

**Results:**

The serum CRP was elevated in 39 patients (H-CRP), while 33 patients were within the normal range (N-CRP). The elevated CRP was associated with Lymph node metastasis (*p* = 0.003) and tumor size (*p* = 0.004). The median survival time after recurrence was significantly worse in the H-CRP group than N-CRP group (6.5 months vs. 11.5 months, *p* = 0.012). Multivariate analyses identified that elevated CRP level (HR=2.325, *p* < 0.001), time to recurrence (HR = 0.466, *p*=0.033), and the follow-up treatment (HR = 2.650, *p*=0.001) were independent prognostic factors.

**Conclusions:**

High serum CRP level was associated with aggressive pathological features, was an independent poor prognostic factors for RGC, which might be a potential prognostic marker for RGC patients.

## INTRODUCTION

As the fourth most common cancer worldwide, the long-term outcome of gastric cancer (GC) remains poor [1–3]. For the GC patients who underwent surgery, recurrence was identified as one of the most important factors for prognosis [4]. More than 60% GC patients were in the advanced stage at the initial diagnosis, and the recurrence occurred in about 62% GC patients who received surgery [5–7]. Numerous studies have investigated the predictive factors, recurrence patterns and the therapy for RGC [8–11]. Unfortunately, no precisely predictive factors were identified for RGC patients. What's worse, no effective nor standard therapy has been established for RGC presently [12]. As the prognosis of RGC remains clinically poor, it is urgent to explore the novel biomarker which could predict early recurrence and prognosis precisely.

Many nonspecific serum inflammatory markers were found playing an important role in tumor progression and prognosis. C-reactive protein (CRP), one of the inflammatory markers, was found its up-regulation was associated with poor prognosis in small cell lung cancer, osteosarcoma, prostate cancer and colorectal cancer [13–16]. Recently, one study found CRP was associated with tumor size in NSCLC [17]. What was more, the other retrospective study reported that high CRP level was associated with poor prognosis of advanced-stage NSCLC patients treated with erlotinib [18]. However, only limited published reports showed the role of inflammatory factors in the RGC.

Based on the above consideration, we measured the CRP level, collected the clinical and survival information, explored the association between serum CRP and clinical-pathological factors, and investigated its prognostic value in RGC patients.

## MATERIALS AND METHODS

### Patients and information collection

72 RGC patients who received treatment from January 2005 to May 2008 were enrolled in this study. Patients with concurrent infection or incomplete follow-up information were excluded. The OS was defined as time from the date of diagnosis to the date of death or last visit. This study was approved by the Research Ethics Committee of First Teaching Hospital of Tianjin University of Traditional Chinese Medicine, China. Written informed consents were obtained from the patients before participating in this study.

Serum CRP level was measured from peripheral venous blood samples. All the patients were divided into high CRP group (H-CRP; >0.8 mg/dL) and normal CRP group(N-CRP; ≤0.8 mg/dL) according to the serum CRP level and previous study [13]. The clinical-pathological information was reviewed from the database of our hospital, and the survival data was collected from clinic visit or family contact. The response evaluation was performed according to the Response Evaluation Criteria in Solid Tumors Guidelines.

### Statistical analysis

The statistical analyses were performed using SPSS version 18.0 (SPSS, Chicago, IL, United States). Continuous variables were described using mean±standard deviation; the survival was compared through the Kaplan–Meier method and log-rank tests. Furthermore, the prognostic role of the CRP was identified by the Multivariate analyses. Significance was defined as *p*-Values< 0.05.

## RESULTS

### Patient characteristics

Among the 72 RGC patients, 9 patients had at least two recurrence patterns concurrently. Of all the RGC patients, 10 patients (13.9%) received a total gastrectomy, and 50 patients (84.7%) received adjuvant chemotherapy according to NCCN guideline. As the Table 1 and Table 2 showed, the peritoneal seeding was the most common recurrence pattern. The clinical and pathological factors were shown in Table 1. 55 patients were male; the average age was 60.0 years, and 34 patients were well differentiated. The median follow-up time for all RGC patients was 55 months.

**Table 1:** The association between CRP and Clinicopathological characteristics (*n* = 72)

	Number	H-CRP group (*n* = 39)	N-CRP group (*n* = 33)	*P* value
Age (years)				0.351
Mean		61.3±9.7	57.9±6.6	
Gender (%)				0.654
Male	55	35 (89.7%)	20 (60.1%)	
Female	17	4 (10.3%)	13 (39.9%)	
Tumor Size (cm)				0.004
<5	22	5 (12.8%)	17 (51.5%)	
≥5	50	34 (87.2%)	16 (49.5%)	
Location of tumor				0.221
Lower	28	15 (38.5%)	13 (39.4%)	
Middle	8	5 (12.8%)	3 (9.1%)	
Upper	36	19 (48.7%)	17 (51.5%)	
Lymph node metastasis				0.003
No	19	10 (25.6%)	9(27.3%)	
Yes	29	22 (56.4%)	7 (21.2%)	
Histology				0.436
Highly/moderately differentiated	34	18 (72.8%)	16 (79.2%)	
Low/Undifferentiated	38	21 (27.2%)	17 (20.8%)	
Patterns of Recurrence				0.386
Locoregional recurrence	18	10(25.6%)	8(24.2%)	
Peritoneal seeding	30	18(46.2%)	12(36.4%)	
Hematogenous recurrence	15	6(15.4%)	9(27.3%)	
Others*	9	5(12.8%)	4(12.1%)	

* The patterns of recurrence involved at least two patterns

**Table 2:** : Recurrence patterns of RGC patients (*n* = 72)

	Number	Percentage
Locoregional recurrence (*n* = 22)		
Remnant stomach	7	31.9%
Gastric bed	3	13.6%
Anastomosis	3	13.6%
Lymph nodes	9	40.9%
Peritoneal seeding (*n* = 31)	31	100%
Hematogenous recurrence (*n* = 19)		
Liver	11	57.9%
Lung	4	21.1%
Bone	2	10.4%
Brain	1	5.3%
Abdominal wall	1	5.3%

### Association between CRP and clinical-pathological factors

There were 39 patients (54.2%) with elevated CRP level, while 33 patients (45.8%) were within the normal range. The mean concentration of serum CRP was 8.8±0.5 mg/dL in the H-CRP group, while 0.4±0.1 mg/dL in the N-CRP group. We further compared the clinical-pathological factors based on the CRP level. As the Table 1 showed, the high CRP level was associated with larger tumor size (*p* = 0.004) and more Lymph node metastasis (*p* = 0.003). In general, the RGC patients with elevated CRP seemed to have more aggressive pathological features.

### Survival outcomes

**Figure 1:**
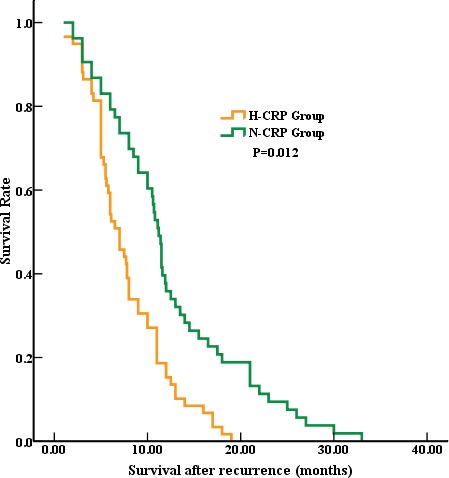
Survival after recurrence in RGC patients The median survival time after recurrence was significantly worse in the H-CRP group compared to N-CRP group (6.5 months vs. 11.5 months, *p* = 0.012).

In our study, the median survival time after recurrence was 10.3 months. The further Kaplan–Meier method and log-rank tests showed that the long-term outcome of H-CRP RGC patients was significantly worse than those RGC patients with normal CRP level (Survival, 11.5 months vs. 6.5 months, *p* = 0.012), these result was showed in Figure. 1.

Cox proportional hazards model was further conducted to identify whether CRP was an independent prognostic factor for survival. As the Table 3 showed, the univariate analysis identified the CRP (*p* = 0.012), Lymph node metastasis (*p* = 0.021), time to recurrence (*p* = 0.001), and the follow-up treatment (*p* = 0.011) were prognostic factors. After multivariate analysis, high CRP level (HR = 2.235, 95% CI, 1.159-2.687; *p* < 0.001), the follow-up treatment (HR = 2.650, 95% CI, 1.608-3.674; *p* = 0.001) and time to recurrence (HR = 0.466, 95% CI, 0.217-0.701; *p* = 0.033) were confirmed as independent markers for RGC survival.

**Table 3:** Univariate and multivariate analysis of factors influencing OS (*n* =72)

Variable	Univariate analysis	Multivariate Cox regression		
	*p*-Value	HR	95% CI	*p-*Value
Age (years)	0.332	−	−	−
CRP	0.012	2.235	1.159-2.687	<0.001
Lymph node metastasis	0.021	1.655	1.307–1.987	0.124
Time to recurrence	0.001	0.466	0.217–0.701	0.033
Tumor size	0.055	−	−	−
The follow-up treatment	0.011	2.650	1.608–3.674	0.001

HR hazard ratio, CI confidence interval.

## DISCUSSION

This report explored the prognostic role of serum CRP in RGC patients. Our data showed that the elevated CRP was associated with more aggressive pathological features. Further analyses identified that high serum CRP level was associated with poor outcome, which might be potential maker defining the prognosis of RGC patients.

The GC recurrence occurred most within 2 years after resection, which resulted in poor prognosis for RGC [5–7]. Many basic and clinical studies focused on the recurrence of GC, but the meaningful result, such as accurate predictors, effective drugs, standard treatment, et al, were still not sufficient. That's to say, it is still urgent to find novel biomarkers and establish effective therapies to improve the prognosis of RGC.

As the previous studies reported, the elevated serum CRP was associated with poor survival in kinds of solid tumors [19–24]. Recently, Dr. Shao and her colleague found that the high CRP level was associated with the poor outcome for combined small cell lung cancer [13]. In the present study, we first investigated the prognostic significance of serum CRP in RGC. Our data identified that RGC patients with higher CRP level had worse survival time compared to N-CRP group (6.5 months vs. 11.5 months, respectively; *p* = 0.012) (Showed in Fig. 1). This result was consistent with previous studies. In addition, in the present study, the multivariate analysis confirmed that the elevated CRP was an independent poor prognostic factor for RGC, which reinforced the prognostic value of CRP in solid tumors. At last, consistent with the other previous studies, we re-confirmed that the follow-up treatment (HR = 2.650, 95% CI, 1.608-3.674; *p* = 0.001), time to recurrence (HR = 0.466, 95% CI, 0.217-0.701; *p* = 0.033) were independent prognosis factors for RGC.

Recently, some studies reported the association between CRP and clinical-pathological factors. For the resected NSCLC, Dr. Lee and his team found that the higher CRP level was associated with more lymph vascular invasion [17]. And one study from Japan showed that the patients with elevated CRP had larger tumor size in hepatocellular carcinoma [20]. One systematic review summarized that CRP was an important biomarker for prognosis and treatment response in kinds of solid tumors [25]. In consistent, our data also showed that the RGC patients with higher CRP level had larger tumor size (*p* = 0.004) and more Lymph node metastasis (*p* = 0.003). All these findings indicated that the elevated CRP level was associated with aggressive pathological parameters.

Although this study had some shortcomings such as small size, single-center study and retrospective design, CRP still has its clinical significance because of its simple, easy detection and accurate prediction. Better designed large scale and multicenter studies should be conducted to confirm our result.

## CONCLUSIONS

The higher serum CRP was associated with aggressive clinical-pathological features resulting in worse long-term outcome in RGC patients. Our study confirmed that the elevated CRP was an independent prognostic factor, which might be potential treatment target for RGC.

## References

[1] Matsukuma A, Furusawa M, Tomoda H, Seo Y (1996). A clinicopathological study of asymptomatic gastric cancer. Br J Cancer.

[2] Liu C, Zhang R, Lu Y, Li H, Lu P, Yao F, Jin F, Xu H, Wang S, Chen J (2010). Prognostic role of lymphatic vessel invasion in early gastric cancer: a retrospective study of 188 cases. Surg Oncol.

[3] Parkin DM, Bray F, Ferlay J, Pisani P (2005). Global cancer statistics, 2002. CA Cancer J Clin.

[4] Xu AM, Huang L, Zhu L, Wei ZJ (2014). Significance of peripheral neutrophil-lymphocyte ratio among gastric cancer patients and construction of a treatment-predictive model: a study based on 1131 cases. Am J Cancer Res.

[5] Buzzoni R, Bajetta E, Di Bartolomeo M, Miceli R, Beretta E, Ferrario E, Mariani L (2006). Pathological features as predictors of recurrence after radical resection of gastric cancer. Br J Surg.

[6] Cidon EU (2010). Gastric cancer and the search for a good prognostic classification: a challenge. Clin Exp Gastroenterol.

[7] Xu DZ, Geng QR, Long ZJ, Zhan YQ, Li W, Zhou ZW, Chen YB, Sun XW, Chen G, Liu Q (2009). Positive lymph node ratio is an independent prognostic factor in gastric cancer after d2 resection regardless of the examined number of lymph nodes. Ann Surg Oncol.

[8] Bilici A, Selcukbiricik F (2015). Prognostic significance of the recurrence pattern and risk factors for recurrence in patients with proximal gastric cancer who underwent curative gastrectomy. Tumour Biol.

[9] Bilici A, Selcukbiricik F (2015). Prognostic significance of the recurrence pattern and risk factors for recurrence in patients with proximal gastric cancer who underwent curative gastrectomy. Tumour Biol.

[10] Li Z, Zhang D, Zhang H, Miao Z, Tang Y, Sun G, Dai D (2014). Prediction of peritoneal recurrence by the mRNA level of CEA and MMP-7 in peritoneal lavage of gastric cancer patients. Tumour Biol.

[11] Kong F, Qi Y, Liu H, Gao F, Yang P, Li Y, Jia Y (2015). Surgery combined with chemotherapy for recurrent gastric cancer achieves better long-term prognosis. Clin Transl Oncol.

[12] Li JH, Zhang SW, Liu J, Shao MZ, Chen L (2012). Review of clinical investigation on recurrence of gastric cancer following curative resection. Chin Med J (Engl).

[13] Shao N, Cai Q (2015). High pretreatment serum C-reactive protein level predicts a poor prognosis for combined small-cell lung cancer. Tumour Biol.

[14] Xu L, Zhao Q, Huang S, Li S, Wang J, Li Q (2015). Serum C-reactive protein acted as a prognostic biomarker for overall survival in metastatic prostate cancer patients. Tumour Biol.

[15] Li X, Tian F, Wang F, Li Y (2015). Serum C-reactive protein and overall survival of patients with osteosarcoma. Tumour Biol.

[16] Fujita T (2007). Preoperative but not postoperative systemic inflammatory response correlates with survival in colorectal cancer (Br J Surg 2007; 94: 1028-1032). Br J Surg.

[17] Lee JG, Cho BC, Bae MK, Lee CY, Park IK, Kim DJ, Ahn SV, Chung KY (2009). Preoperative C-reactive protein levels are associated with tumor size and lymphovascular invasion in resected non-small cell lung cancer. Lung Cancer.

[18] Fiala O, Pesek M, Finek J, Topolcan O, Racek J, Minarik M, Benesova L, Bortlicek Z, Poprach A, Buchler T (2015). High serum level of C-reactive protein is associated with worse outcome of patients with advanced-stage NSCLC treated with erlotinib. Tumour Biol.

[19] Kasymjanova G, MacDonald N, Agulnik JS, Cohen V, Pepe C, Kreisman H, Sharma R, Small D (2010). The predictive value of pre-treatment inflammatory markers in advanced non-small-cell lung cancer. Curr Oncol.

[20] Hashimoto K, Ikeda Y, Korenaga D, Tanoue K, Hamatake M, Kawasaki K, Yamaoka T, Iwatani Y, Akazawa K, Takenaka K (2005). The impact of preoperative serum C-reactive protein on the prognosis of patients with hepatocellular carcinoma. Cancer.

[21] Hefler LA, Concin N, Hofstetter G, Marth C, Mustea A, Sehouli J, Zeillinger R, Leipold H, Lass H, Grimm C, Tempfer CB, Reinthaller A (2008). Serum C-reactive protein as independent prognostic variable in patients with ovarian cancer. Clin Cancer Res.

[22] Karakiewicz PI, Hutterer GC, Trinh QD, Jeldres C, Perrotte P, Gallina A, Tostain J, Patard JJ (2007). C-reactive protein is an informative predictor of renal cell carcinoma-specific mortality: a European study of 313 patients. Cancer.

[23] Shimada H, Nabeya Y, Okazumi S, Matsubara H, Shiratori T, Aoki T, Sugaya M, Miyazawa Y, Hayashi H, Miyazaki S, Ochiai T (2003). Elevation of preoperative serum C-reactive protein level is related to poor prognosis in esophageal squamous cell carcinoma. J Surg Oncol.

[24] Crozier JE, McKee RF, McArdle CS, Angerson WJ, Anderson JH, Horgan PG, McMillan DC (2007). Preoperative but not postoperative systemic inflammatory response correlates with survival in colorectal cancer. Br J Surg.

[25] Shrotriya S, Walsh D, Bennani-Baiti N, Thomas S, Lorton C (2015). C-Reactive Protein Is an Important Biomarker for Prognosis Tumor Recurrence and Treatment Response in Adult Solid Tumors: A Systematic Review. PLoS One.

